# Are telephone consultations here to stay in rheumatology?

**DOI:** 10.1093/rap/rkaa071

**Published:** 2020-12-16

**Authors:** Sabrina R Raizada, Natasha Cleaton, James Bateman, Diarmuid M Mulherin, Nick Barkham

**Affiliations:** Rheumatology Department, The Royal Wolverhampton NHS Trust, Wolverhampton, UK

**Keywords:** telephone consultations, rheumatology, face-to-face appointments

## Abstract

**Objectives:**

During the COVID-19 pandemic, face-to-face rheumatology follow-up appointments were mostly replaced with telephone or virtual consultations in order to protect vulnerable patients. We aimed to investigate the perspectives of rheumatology patients on the use of telephone consultations compared with the traditional face-to-face consultation.

**Methods:**

We carried out a retrospective survey of all rheumatology follow-up patients at the Royal Wolverhampton Trust who had received a telephone consultation from a rheumatology consultant during a 4-week period via an online survey tool.

**Results:**

Surveys were distributed to 1213 patients, of whom 336 (27.7%) responded, and 306 (91.1%) patients completed all components of the survey. Overall, an equal number of patients would prefer telephone clinics or face-to-face consultations for their next routine appointment. When divided by age group, the majority who preferred the telephone clinics were <50 years old [χ^2^ (d.f. = 3) = 10.075, *P* = 0.018]. Prevalence of a smartphone was higher among younger patients (<50 years old: 46 of 47, 97.9%) than among older patients (≥50 years old: 209 of 259, 80.7%) [χ^2^ (d.f. = 3) = 20.919, *P* < 0.001]. More patients reported that they would prefer a telephone call for urgent advice (168, 54.9%).

**Conclusion:**

Most patients interviewed were happy with their routine face-to-face appointment being switched to a telephone consultation. Of those interviewed, patients >50 years old were less likely than their younger counterparts to want telephone consultations in place of face-to-face appointments. Most patients in our study would prefer a telephone consultation for urgent advice. We must ensure that older patients and those in vulnerable groups who value in-person contact are not excluded. Telephone clinics in some form are here to stay in rheumatology for the foreseeable future.

**Key messages**

- Most patients are happy with telephone consultations rather than face-to-face appointments.
- Older patients are less likely to want telephone consultations in place of face-to-face appointments.
- Most patients would prefer a telephone consultation for urgent advice.

## Introduction

Patients with rheumatological disease have traditionally been followed up in secondary care with face-to-face appointments. In 2018, the National Health Service (NHS) in the UK was responsible for 5% of all road traffic, detrimentally impacting the environment and contributing to the conclusion that the current model is unsustainable and no longer fit for purpose [1]. Over the last 10 years, there has been a growing interest in the use of virtual consultation between clinicians and patients, and there is trial evidence to suggest that this is both safe and effective in selected patients [2, 3]. Telephone advice lines are used by clinical nurse specialists for acute care, and telephone follow-up has been used successfully in a study in Denmark to achieve disease control in RA patients with low disease activity [4]. There has been discussion about redesigning rheumatology services to enable more telephone and virtual consultations for routine care, but little is known about the patient perspective regarding this.

Owing to a combination of immunosuppression, underlying disease and co-morbidities, a significant proportion of patients with rheumatic disease are considered vulnerable and at increased risk from COVID-19 [5]. In view of this, and as advised by evolving national guidance produced by the National Institute for Health and Care Excellence (NICE) and the British Society for Rheumatology, we have adapted our services during the pandemic to minimize risk to our patients. One such adaptation has been that face-to-face appointments have largely been suspended, instead relying on telephone or virtual consultations followed by a face-to-face appointment if necessary [6].

## Methods

We initially contacted our local patient participation group to obtain members’ perspective on the use of telephone consultations in comparison to the usual face-to-face consultations. Of the 42 responses via email from patients with RA, all respondents felt that although telephone consultations were suitable, face-to-face consultations were preferred.

In response to this feedback, we designed and piloted a 5-min, 12-item questionnaire (SurveyMonkey, 2020) with our patient participation group as part of our on-going service evaluation and development. The survey questions are detailed in Supplementary Data S1, available at *Rheumatology Advances in Practice* online. We contacted all rheumatology follow-up patients at the Royal Wolverhampton Trust (RWT) who had received a telephone consultation from a rheumatology consultant during a 4-week period (from 11 May to 4 June 2020). Surveys were distributed to patients using a recently described mobile phone short message service (SMS) messaging methodology via our SMS provider (Healthcare Communications-UK) [7].

Patients surveyed were asked to self-report a range of metrics, including demographics, diagnosis and medication, in addition to specific questions regarding the quality of their recent consultation, thoughts on future consultations, access to smartphone technology and how they would like to be contacted by the department in the future.

Data were collected in an anonymized format and analysed using SPSS v.26. Initially, data were assessed for the entire cohort; subsequently, patients were divided into four age groups, as reported in the survey.

## Results

During the 4-week period, 1213 rheumatology follow-up patients with validated mobile numbers had a telephone consultation with a consultant rheumatologist at RWT. These 1213 patients had a mean age of 59.0 years (s.d. 14.3 years); 1018 (83.9%) were Caucasian, 145 (12.0%) were Black, Asian or minority ethnic group, and for 50 (4.1%) ethnicity was not recorded (Supplementary Table S1, available at *Rheumatology Advances in Practice* online). Responses were received from 336 of 1213 patients (27.7%), and of these, 306 (91.1%) had completed all components of the survey. Only complete responders were included in the analysis.

The number of complete survey responders in each age category included: 1 (0.3%) patient aged 16–29 years, 46 (15.0%) aged 30–49 years, 180 (58.8%) aged 50–69 years and 79 (25.8%) aged ≥70 years. Complete responders were mostly female [227 of 306 (74.2%)]. The majority had a diagnosis of RA [171 (55.9%)], PsA [37 (12.1%)] or CTD [19 (6.2%)]. Seven (2.3%) had osteoporosis and 45 (14.7%) classified themselves as being in the ‘other’ diagnostic category (Supplementary Table S1, available at *Rheumatology Advances in Practice* online). Seventy-eight (25.5%) were taking a biological treatment, 174 (56.9%) were taking conventional DMARDs and 57 (18.6%) were taking glucocorticoids as part of their rheumatic disease management (Supplementary Table S1, available at *Rheumatology Advances in Practice* online).

When asked to review their recent telephone consultation, 265 of 306 (86.6%) agreed or strongly agreed that they were satisfied with the telephone consultation, 27 (8.8%) disagreed or strongly disagreed and 14 (4.6%) were neutral (Table 1). The majority [249 (81.4%)] agreed or strongly agreed that they were pleased to have a telephone consultation rather than face-to-face owing to the current pandemic, 32 (10.5%) disagreed or strongly disagreed and 25 (8.2%) remained neutral. Far fewer [48 (15.7%)] agreed or strongly agreed that they would prefer a video consultation, 110 (36.0%) disagreed or strongly disagreed and the majority [148 (48.4%)] were neutral. Most patients felt that the duration of the consultation was satisfactory: 267 (87.3%) agreed or strongly agreed, 23 (7.5%) disagreed or strongly disagreed and 16 (5.2%) felt neutral. Most [277 (90.5%)] agreed or strongly agreed they had ‘no difficulty hearing what was said and my doctor could hear me clearly’; 20 (6.5%) disagreed or strongly disagreed and 9 (2.9%) were neutral. The majority [266 of 306 (86.9%)] agreed or strongly agreed that they felt all their questions were answered, whereas 29 (9.5%) disagreed or strongly disagreed and 11 (3.6%) felt neutral (Table 1).

**Table 1:** Satisfaction with the telephone consultation

Response	Strongly agree	Agree	Neutral	Disagree	Strongly disagree
All of my questions were answered, *n* (%)	144 (47.1)	122 (39.9)	11 (3.6)	5 (1.6)	24 (7.8)
I was satisfied with the telephone consultation, *n* (%)	158 (51.6)	107 (35.0)	14 (4.6)	5 (1.6)	22 (7.2)
I felt pleased to have a chance to have a ’phone consultation rather than face to face because of COVID, *n* (%)	166 (54.3)	83 (27.1)	25 (8.2)	10 (3.3)	22 (7.2)
The length of the consultation was satisfactory, *n* (%)	142 (46.4)	125 (40.9)	16 (5.2)	6 (2.0)	17 (5.6)
I had no difficulty hearing what was said, and my doctor could hear me clearly, *n* (%)	165 (53.9)	112 (36.6)	9 (3.0)	3 (1.0)	17 (5.6)
I would have preferred a ‘video’ consultation rather than a telephone consultation, *n* (%)	13 (4.3)	35 (11.4)	148 (48.4)	82 (26.8)	28 (9.2)

We found that 175 of 306 (57.2%) reported they would be happy for their next routine appointment to be a telephone clinic. A significantly higher proportion of patients younger than 50 years (16–29 years: 0 of 1, 0%; 30–49 years: 34 of 46, 73.9%) preferred telephone consultations when compared with older patients (50–69 years: 104 of 180, 57.8%; >70 years: 37 of 79, 46.8%) [χ^2^ (d.f. = 3) = 10.075, *P* = 0.018; Table 2].

**Table 2:** Preference of consultation mode: responses by age

Response	Overall *n* = 306	16–29 years old *n* = 1	30–49 years old *n* = 46	50–69 years old *n* = 180	>70 years old *n* = 79	χ^2^ (d.f.)	*P*-value
I would be happy with my routine/regular face-to-face clinic appointment being switched to telephone clinic, *n* (%)							
Yes	175 (57.2)	0 (0)	34 (73.9)	104 (57.8)	37 (46.8)	10.075^*^ (3)	0.018
No	131 (42.8)	1 (100)	12 (26.1)	76 (42.2)	42 (53.2)		
Do you have a smartphone which could be used to make a video call? *n* (%)							
Yes	255 (83.3)	1 (100)	45 (97.8)	155 (86.1)	54 (68.4)	20.919^*^ (3)	<0.001
No	51 (16.7)	0 (0)	1 (2.2)	25 (13.9)	25 (31.7)		
How would you prefer to be notified about your future telephone appointments? *n* (%)							
Text	143 (46.7)	0 (0)	26 (56.5)	80 (44.4)	37 (46.8)	3.947 (6)	0.684
Letter	38 (12.4)	0 (0)	4 (8.7)	25 (13.9)	9 (11.4)		
Both text and letter	125 (40.9)	1 (100)	16 (34.8)	75 (41.7)	33 (41.8)		
Preferred routine contact with my rheumatology doctor would be, *n* (%)							
Face to face	144 (47.1)	1 (100)	15 (32.6)	80 (44.4)	48 (60.8)	11.081 (6)	0.086
Telephone consultation	131 (48.8)	0 (0)	25 (54.4)	81 (45.0)	25 (31.7)		
Video call	31 (10.1)	0 (0)	6 (13.0)	19 (10.6)	6 (8.0)		
Preferred emergency contact would be, *n* (%)							
Face to face	104 (34.0)	0 (0)	11 (23.9)	61 (33.9)	32 (40.5)	12.138 (6)	0.059
Telephone consultation	168 (54.9)	0 (0)	29 (63.0)	98 (54.4)	41 (51.9)		
Video call	34 (11.1)	1 (100)	6 (13.0)	21 (11.7)	6 (7.6)		

* Significant result of P-values.

Again, prevalence of a smartphone was higher among younger patients (16–29 years: 1 of 1, 100%; 30–49 years: 45 of 46, 97.8%) than among those in the older cohort (50–69 years: 155 of 180, 86.1%; >70 years: 54 of 79, 68.4%) [χ^2^ (d.f. = 3) = 20.919, *P* < 0.001; Table 2].

There was no significant difference between the proportion of patients in different diagnostic groups (autoimmune rheumatic disease *vs* CTD/vasculitis *vs* other) who would be happy for their next routine appointment to be a telephone clinic [χ^2^ (d.f. = 2) = 1.070, *P* = 0.590] and the proportion of patients in each group with access to a smartphone [χ^2^ (d.f. = 2) = 4.035, *P* = 0.133].

Most (143 of 306, 46.7%) reported that they would prefer to be contacted about future appointments via text message; 125 (40.9%) would prefer both text message and a letter; far fewer would prefer only a letter contact (38, 12.4%). There was no significant difference in the preferred method of contact between patients in different age groups [χ^2^ (d.f. = 6) = 3.947, *P* = 0.684; Table 2].

The proportion of patients who reported that they would prefer their routine contact with their rheumatology doctor to be a ‘telephone consult’ was 131 of 306 (42.8%), similar to the proportion reporting that they would prefer ‘face-to-face’ 144 of 306 (47.1%), whereas only 31 of 306 (10.1%) reported they would have preferred a ‘video call’; this did not differ significantly between age groups [χ^2^ (d.f. = 6) = 11.081, *P* = 0.86]. More patients reported that they would prefer a telephone call for urgent advice (168, 54.9%), whereas video call was least favoured (34, 11.1%); again, this did not differ significantly across different age groups [χ^2^ (d.f. = 6) = 12.138, *P* = 0.059; Table 2].

## Discussion

These findings provide a review of telephone consultations and present insight into the perspectives of patients on the use of telephone consultations in place of face-to-face consultations provided as part of an important safety measure taken during the COVID-19 pandemic. Importantly, these data contribute to our understanding of our patients’ views in adopting telephone consultations in place of face-to-face appointments on a longer-term basis, as set out in the NHS long-term plan [8].

The smartphone-based methodology used in the present study was accepted by this population, supported by our response rate, which was consistent with other surveys, and illustrating its potential role during a public health emergency [7, 9]. The case-mix distribution in this survey was representative of the patients we normally see in our face-to-face follow-up rheumatology clinics, adding strength to our findings [10].

Overall, the vast majority of patients (86.6%) were satisfied with the telephone consultation they had as a replacement for their face-to-face appointment during the pandemic, and most (54.9%) would prefer a telephone consultation rather than face-to-face or video call appointment for urgent advice. We did not have data on the proportion of patients who had accessed nurse-led telephone services. However, it is likely that several of these patients would have accessed this service before the pandemic, and the response they received then might have influenced their attitude towards telephone consultations. However, although around half the patients would be happy to exchange their routine face-to-face appointment for a telephone consultation, there was a significant difference in the number of patients <50 years (73.9%) and those aged ≥50 years (54.4%) who would be happy with the telephone consultation rather than face-to-face.

These findings highlight that patients in older age groups are less likely to want telephone consultations in place of face-to-face appointments as demonstrated by the pilot survey. The age population of our patient participation group is generally older than our patient follow-up cohort, and all of these patients preferred face-to-face consultation. The patients in this group had not experienced a telephone consultation, and their views regarding telephone consultations were less favourable than the responses from patients who had received a telephone consultation. Despite the widespread uptake of smartphones, there was relatively limited enthusiasm for video-based consultations, and a cultural shift might be required before telemedicine is accepted. A significant proportion of our patients were Black, Asian or minority ethnic group, and their first language might not be English. Their views might vary on face-to-face consultations, because our earlier work has demonstrated differing views in this population [11].

There was a significant difference between younger patients (<50 years) and patients aged ≥50 years who had access to a mobile smartphone. In addition to negative attitudes generally held by older adults towards smartphones, those in this age group might suffer more with poor dexterity than younger patients, causing practical issues in using a smartphone [12]. Older patients with longer-standing inflammatory arthritis tend to have more joint deformity [13]. These findings indicate that those in older generations might struggle if consultations relied solely on the use of smartphone technology. However, engagement by the elderly with smartphone technology has massively increased throughout lockdown during the pandemic, and this might enable increased acceptance and uptake in the future for telephone consultations [14].

Social isolation in the elderly might account for some of the differences in responses between patients across different age groups. Social isolation itself is associated with adverse health consequences [15], exemplified by recent study data that found that ‘social shielding’, strict social isolation measures adopted during the COVID pandemic, adversely impacted the mental health of patients in this population [16]. Most of our follow-up cohorts have chronic arthritis, and they form strong relationships with other patients and members of staff whom they meet on a regular basis in secondary care. Moving away from face-to-face consultations would remove this valued contact for these patients.

This survey was sent to all rheumatology follow-up patients at RWT who had received a telephone consultation from a rheumatology consultant during a 4-week period. The majority of these patients would have had an inflammatory arthritis. Previous work has demonstrated that it is not possible to use traditional measures of disease activity, such as the DAS28, in virtual consultations because the swollen count tends to be overestimated [17]. Other studies have used specific outcome measures, such as the Flare-RA instrument, and have found that telephone consultations are useful for patients with low disease activity or remission, where physical examination is not so crucial [4]. Telephone consultations would be useful for follow-up of conditions such as SpA, where outcome measures such as BASDAI do not require a clinical examination. All patients surveyed were follow-up patients; reviewing new patients in rheumatology via telephone would have additional limitations owing to the importance of a clinical examination in diagnosis.

There are several limitations to this study; the difficulties associated with collecting large amounts of data across complex datasets have been recognized [18]. The SMS-based distribution of this survey excludes patients without access to smartphone or internet technology. It also excludes patients who do not favour virtual communication and are less inclined to be involved in the study. A previous study looking at telehealth demonstrated that older patients were less likely to take part in the study [4]. Therefore, the number of older patients preferring face-to-face consultations might be higher than reported, because older patients who are not keen on virtual consultations are probably less likely to participate in online surveys. We might not reach elderly patients and other vulnerable groups in our population owing to the poorer health care and digital literacy associated with social deprivation [19, 20].

The COVID-19 pandemic has precipitated a wholescale adoption of remote consultation on a scale never previously envisaged, and there can be no going back to the previous model. Most patients are happy with telephone consultations, at least in the short term, but we must ensure that older patients and those in vulnerable groups who value and do not wish to lose in-person contact are not excluded. Telephone clinics in some form are here to stay in rheumatology for the foreseeable future.

*Funding*: No specific funding was received from any funding agency in the public, commercial or not-for-profit sectors to carry out the work described in this manuscript.

*Disclosure statement*: The authors have declared no conflicts of interest.

## Supplementary data

Supplementary data are available at *Rheumatology Advances in Practice* online.

## Supplementary Material

rkaa071_Supplementary_Data
