# Supplementary material for: Are telephone consultations here to stay in rheumatology?
Source: Rheumatol Adv Pract. 2020 Dec 16;5(1):rkaa071. doi: 10.1093/rap/rkaa071 (PMC7798592; doi:10.1093/rap/rkaa071)
Supplement: rkaa071_Supplementary_Data [file rkaa071_supplementary_data.zip › RAP 20-094 Supplementary Table S1.docx]

**Supplementary Table S1: Demographic characteristics of patients included in the survey.**

| **All Patients that received SMS invite** | 1213 |
| --- | --- |
| **Age, Mean** [SD] | 59.03 [14.3] |
| **Ethnicity**, n (%)  Caucasian, n (%)  BAME, n (%)  Not Stated, n (%) | 1018 (83.9%)  145 (12.0%)  50 (4.11%) |
| **Survey Responders**, n (%) | 306 (25.2%) |
| **Gender, n (%)**  Male  Female | 79 (25.8%)  227 (74.2%) |
| **Age year categories, n [SD] (%)**  16 – 29  30 – 49  50 – 69  >70 | 1[0.06] (0.3%)  46 [0.36] (15.0%)  180 [0.49] (58.8%)  79 [0.44] (25.8%) |
| **Diagnosis, n (%)**  RA  AS  PsA  Gout  SLE  Vasculitis  PMR  Osteoporosis  Other | 171 (55.9%)  7 (2.3%)  37 (12.1%)  1 (0.3%)  19 (6.2%)  6 (2.0%)  13 (4.2%)  7 (2.3%)  45 (14.7%) |
| **Medication, n (%)**  Painkillers/anti-inflammatory  DMARDs  Biologics  Steroids | 136 (44.4%)  174 (56.9%)  78 (25.5%)  57 (18.6%) |

SMS, Short Message Service; SD, standard deviation; BAME, black, Asian, and minority ethnic; RA, rheumatoid arthritis; AS, ankylosing spondylitis; PsA, psoriatic arthritis; SLE, systemic lupus erythematosus; PMR, polymyalgia rheumatic; DMARDs, disease-modifying antirheumatic drugs
