# Supplementary material for: Are telephone consultations here to stay in rheumatology?
Source: Rheumatol Adv Pract. 2020 Dec 16;5(1):rkaa071. doi: 10.1093/rap/rkaa071 (PMC7798592; doi:10.1093/rap/rkaa071)
Supplement: rkaa071_Supplementary_Data [file rkaa071_supplementary_data.zip › Supplementary Data S1. Survey questions.docx]

1. **Please enter your gender**
2. **Please enter your age**
3. **Please choose the diagnosis that best fits your case:**

- Rheumatoid arthritis
- Ankylosing spondylitis
- Psoriatic arthritis
- Gout
- SLE/ Sjogrens/ Connective tissue disease
- Vasculitis
- Polymyalgia rheumatica
- Osteoporosis
- Other (please specify) [please do not include any personal information]

1. **Please tell us what rheumatology medication you are on: Please choose as many as apply**

- Anti-inflammatory tablets and standard painkillers (paracetamol)
- Disease Modifying Drug tablets (such as methotrexate, sulfasalazine, hydroxychloroquine, leflunomide etc.)
- Biologic drug (such as etanercept, adalumimab, infliximab Rituximab, Abatacept, tocilizumab, baricitinib, tofacitinib, secukinumab)
- Steroid tablets

1. **Please rate your agreement with the following (using this scale):**

| Strongly disagree | Disagree | Neutral | Agree | Strongly Agree |
| --- | --- | --- | --- | --- |

| I was satisfied with the telephone consultation |
| --- |
| All my questions were answered. |
| I felt pleased to have a chance to have a phone consultation rather than face to face because of COVID 19. |
| The length of the consultation was satisfactory. |
| I had no difficulty hearing what was said, and my doctor could hear me clearly. |
| I would have preferred a **'video'** consultation rather than a telephone consultation. |

| 1. **Please think about your next rheumatology appointment (tick your preferred options):**  \|  \| **Face to face consultation (coming to the hospital)** \| **Telephone call consultation** \| **Video call (smartphone) consultation** \| \| --- \| --- \| --- \| --- \| \| **My Preferred routine contact with my rheumatology doctor would be** \|  \|  \|  \| \| **My Preferred emergency  contact with my rheumatology doctor would be** \|  \|  \|  \|  1. **I would be happy with my routine / regular face to face clinic appointment being switched to a telephone clinic appointment**.  - Yes - No |  |  |
| --- | --- | --- | --- | --- | --- | --- | --- | --- | --- | --- | --- | --- | --- | --- |

1. **What happened as a result of the consultation today? ( please choose as many as apply)**

Change in treatment

Advice and reassurance given

Further tests or investigations arranged

Supply of existing medications / prescriptions arranged

Other

1. **Do you have a smartphone which could be used to make a video call?**

Yes

No

1. **How would you prefer to be notified about your future telephone appointments?**

- Text
- Letter
- Both text and letter

1. **Please tell us what worked well? (please do NOT include any personal information, or anything that identifies you)**
2. **What could we improve about consultations like this in the future  (please do NOT include any personal information or anything that identifies you).**
